# Supplementary material for: Appraisal of healthcare students and professionals’ knowledge and intention to educate the public regarding monkeypox (Mpox)
Source: PeerJ. 2025 Apr 4;13:e19162. doi: 10.7717/peerj.19162 (PMC11974541; doi:10.7717/peerj.19162)
Supplement: Supplemental Information 1 [file peerj-13-19162-s001.docx]

Codebook

Age

20-25=0

26-30=1

31-40=2

41-50=3

>50=4

Gender

Male=0

Female=1

Marital status

Single=0

Married=1

Divorced=2

Widow=3

Working status

Student=0

Working=1

Educational level

Student=0

Bachelor=1

Master=2

PhD=3

Field of education

Pharmacy=0

Medicine=1

Dentistry=2

Nursing=3

Physiotherapy=4

Medical laboratory science=5

Awareness

No=0

Not sure=0

Yes=1

Knowledge

No=0

Not sure=0

Yes=1

Monkeypox symptoms usually start within …. of exposure to the virus

Immediately=0

Few days=2

3 weeks=1

Not sure=3

For how many days do symptoms of Monkeypox last

2 - 5 days=0

2 – 4 weeks=1

Very long time=2

Not sure=3

Knowledge Score

Incorrect=0

Correct=1

Intention

5 Likert scale

Strongly Disagree =1

Disagree= 2

Neutral =3

Agree =4

Strongly Agree =5

Scores were inverted for negatively worded items.
